# SHANK3 Genotype Mediates Speech and Language Phenotypes in a Nonclinical Population

**DOI:** 10.1155/2021/6634584

**Published:** 2021-06-03

**Authors:** Christina Manning, Peter L. Hurd, Silven Read, Bernard Crespi

**Affiliations:** ^1^Department of Biological Sciences, Simon Fraser University, Burnaby, BC, Canada; ^2^Department of Psychology and Centre for Neuroscience, University of Alberta, Edmonton, Canada

## Abstract

Mutations affecting the synaptic-scaffold gene SHANK3 represent the most common genetic causes of autism with intellectual disability, accounting for about 1-2% of cases. Rare variants of this gene have also been associated with schizophrenia, and its deletion results in the autistic condition known as Phelan–McDermid syndrome. Despite the importance of SHANK3 as a paradigmatic gene mediating neurodevelopmental disorders, its psychological effects in nonclinical populations have yet to be studied. We genotyped the nonsynonymous, functional SHANK3 SNP rs9616915 in a large population of typical individuals scored for autism spectrum traits (the Autism Quotient, AQ) and schizotypy spectrum traits (the Schizotypal Personality Questionnaire, SPQ-BR). Males, but not females, showed significant genotypic effects for the SPQ-BR subscale associated with speech and language: Odd Speech. These findings, in conjunction with animal model studies showing vocalization and auditory effects of SHANK3 mutations, and studies indicating severe language alterations and speech-associated white matter tract abnormalities in Phelan–McDermid syndrome, suggest that SHANK3 differentially affects the development and expression of human language and speech. Imaging genetic and speech-language studies of typical individuals carrying different genotypes of rs9616915 should provide novel insights into the neurological and psychological bases of speech and language alterations among individuals with SHANK3 mutations and Phelan–McDermid syndrome.

## 1. Introduction

Autism spectrum disorder (ASD) is a highly heritable psychiatric disorder that involves varying degrees of impaired social interaction, restricted interests, repetitive behaviors, and deficits in verbal communication [1]. ASD has multiple causes including common alleles with small effects on risk, rare variants with large deleterious effects, and environmental risk factors [2].

Genes with large effects on risk provide good models for studying autism because their effects can be traced from genetic alterations, to expression or activity differences, and to neurodevelopmental sequelae and autism symptoms. The gene SHANK3 in particular exhibits notable effects on autism risk, with large-effect mutations being responsible for an estimated 1-2% of cases, especially in conjunction with intellectual disability [3, 4].

SHANK3 is located at 22q.13.3 and encodes two protein domains: SRC Homology-3 (SH3), which interacts with adaptor proteins for protein binding, and the ankyrin repeat (ANK), which acts as a scaffold for protein-protein interactions at excitatory glutamatergic synapses [5]. Alterations to SHANK3 influence glutamatergic synapse function through their effects on levels of NMDA and AMPA receptors, impacting dendritic spine number and size [1, 2].

Mutations or deletions of SHANK3 result in Phelan‐McDermid syndrome (PMS), and PMS has been associated with autism, schizophrenia, and bipolar disorder [2, 6, 7]. Four main lines of evidence link SHANK3 with human psychiatric disorders: (1) syndromic effects caused by deletions, (2) large-effect mutations within the gene, (3) common risk alleles, and (4) studies of mouse models.

Phelan–McDermid syndrome is a rare genetic disorder with approximately 2000 cases reported worldwide [8, 9]. The disorder is caused by mutations in SHANK3 or the loss of genes including SHANK3 in the 22q13.3 region, with such deletions varying in size from about 100 kb to 9Mb [8–10]. PMS is characterized by developmental delay, intellectual disability, hypotonia, autistic behaviors, seizures, and delayed or absent speech [7, 10]. About 50% of adolescent or adult PMS patients are diagnosed with bipolar disorder [11] and about 75% are diagnosed with autism, usually as children [8]. The gene SHANK3 is located in the PMS deletion region, and evidence from deletion mapping and case studies of SHANK3 mutations show that haploinsufficiency or disruption of SHANK3 causes the primary cognitive and behavioral symptoms of this syndrome [9, 10].

Duplications of the genomic region including SHANK3 have been reported in case studies of individuals with schizophrenia, anxiety, OCD, intellectual disability, psychosis, ADHD, bipolar disorder, and epilepsy [11–14]. Crespi et al. [13] found that deletions including SHANK3 result in higher autism risk, while duplications including SHANK3 increase the risk of schizophrenia. These findings indicate that SHANK3 gene dosage can mediate the expression of a range of psychiatric conditions.

Large-effect mutations are important in identifying the range of phenotypes that can accompany alterations to SHANK3. Durand et al. [15] studied SHANK3 mutations in three families and determined that frameshifts and translocations were associated with autism. Moessner et al. [3] identified one *de novo* SHANK3 mutation and nine inherited nonsynonymous SHANK3 mutations in a study of 400 autism patients. Similarly, Gauthier et al. [16] identified a *de novo* SHANK3 splice deletion and an inherited missense mutation associated with autism, as well as a nonsense mutation of R1117X and a amissense mutation of R536W related to schizophrenia. Gong et al. [17] identified a heterozygous nonsense mutation Y1015 and an inherited missense mutation A921T of SHANK3 in a population of Chinese patients with intellectual disability. Boccuto et al. [18] found three cases of amino acid deletion causing frameshift mutation in SHANK3 and one case of insertion causing a premature stop codon in patients with speech delay and autism. Waga et al. [19] found four SHANK3 missense mutations, two deletions and two insertions among 128 autism patients, but not in the 228 controls. De Sena Cortabitarte et al. [20] identified five SHANK3 mutations in a population of 500 schizophrenic patients, but not in the 2468 European controls from the Human Genome Project. Numerous studies have thus provided evidence that disruption of SHANK3 is associated with autism and schizophrenia.

SNP studies provide insight into SHANK3 function and how SHANK3 alleles of small effect may contribute to the risks of disorders. Shao et al. [21] studied the rs9616915 SNP in 212 patients and 636 controls in Chinese population and found the TT genotype to be associated with a higher risk of autism, whereas Mashyekhi et al. [22] identified the CT genotype as increasing autism risk in a study of 90 autism patients and 100 controls. Jonsson et al. [23] found no significant association between alleles of rs9616915 and autism in a Swedish population of 12,319 children. Similarly, Qiu et al. [24] identified no link between rs9616915 and autism in a Chinese population of 229 cases and 241 controls. Alleles of the SNP rs76224556 [18] have been associated with autism, while the SNPs rs756638, rs4824116, rs76268556, rs75767639 [24], and rs13331 [25] had no observed impact on autism risk. Bipolar disorder has also been studied due to its frequent occurrence in PMS. A variety of SHANK3 SNPs, including rs9616915, were analyzed, but none were found to be significantly associated with bipolar disorder [26]. Thus, there is mixed evidence on the impact of SNP variation in SHANK3 on autism, while for bipolar disorder, there is no evidence of a significant relationship.

Many studies of mouse models have analyzed the phenotypic effects of SHANK3 alterations. Zhou et al. [27] found that the schizophrenia-related mutation R1117X increased social dominance and allogrooming in mice, while the autism-associated InsG3680 mutation resulted in repetitive behavior and self-injurious grooming. Kouser et al. [28] generated a SHANK3 mutation that deleted exon 21, which led to reduced spatial learning, memory, motor coordination, and social interaction in mice. Peça et al. [29] created a SHANK3 knockout through a mutation in the PDZ domain that caused various degrees of repetitive behaviors, impaired social interaction, and anxiety in mice. The same SHANK3 knockout was also found to increase grooming time and aggressive behaviors, cause learning deficits, and lead to improved pitch discrimination [30]; this latter trait is also commonly found among persons with autism [31]. Han et al. [14] created a SHANK3 duplication in transgenic mice, which resulted in manic-like behavior defined by increased locomotor activity, higher acoustic startle response, and seizures. Mei et al. [32] restored SHANK3 expression in adult knockout mice, which returned repetitive grooming and social interaction to normal levels. Diverse findings from SHANK3 mouse models thus indicate that traits related to communication, autism, and bipolar disorder are impacted by SHANK3 gene alteration.

Alterations to speech and language including absent, simplified, and delayed vocalization are commonly found in both Phelan–McDermid syndrome and autism [9, 33]. Based on these findings, Ponson et al. [34] distinguished PMS as a disorder especially of language and cognitive disability caused by neurophysiological networks differing from those found in autism, although restricted interests and repetitive behavior are also usually still common [8, 9].

Vocalization and response to communication in SHANK3 animal models are of particular interest in the context of language. Ultrasonic vocalizations (USV) of rodents mediate their social communication [35]. Berg et al. [35] observed that rats with reduced expression of SHANK3 had a lower social approach response when exposed to USVs of other mice, and Engineer et al. [36] found that SHANK3 heterozygous rats had weaker auditory responses to human speech sounds compared to controls. Zhou et al. [37] created SHANK3 indels in macaques analogous to InsG3608, a mutation linked to autism in humans. These mutant macaques experienced sleep disturbances, repetitive behaviors, impaired social interactions, and reduced vocalization. These observations indicate notable evidence for associations of SHANK3 expression with communication and vocalization phenotypes in both humans and nonhuman animals.

A large body of work has analyzed SHANK3 in relation to psychiatric disorders and effects in animal models, but no previous study has examined the effects of variation in the gene within healthy, nonclinical human populations. Such studies are useful because they can help to demonstrate the adaptive functions of this gene under nonpathological conditions, which provides insight into the impacts of large-scale alterations.

In this article, we compared autism and schizotypy-related phenotypes to SHANK3 rs9616915 SNP genotypes in a large nonclinical population. To evaluate autism and schizotypal phenotypes, the Autism Spectrum Quotient (AQ) [38] and Schizotypal Personality Questionnaire-Brief Revised (SPQ) [39] were used because, as noted above, there is evidence of previous association of SHANK3 with autism and schizophrenia. The SNP rs9616915 was chosen because of its previously observed functional effects on SHANK3 expression in the hippocampus [26] and because the SNP has previously been associated with autism [21, 22]. The rs9616915 SNP is also of interest because it is polymorphic in both humans and Neanderthals. All other mammals in the UCSC Genome Browser are listed as monomorphic for the C allele, except squirrel monkeys that are monomorphic for T. Given the associations of SHANK3 with PMS and the effects on speech and language in PMS, we predicted that the SHANK3 rs9616915 SNP would affect nonclinical autism or schizotypal phenotypes relevant to PMS, in particular the subscale traits associated with speech and language.

## 2. Methods

This study was approved by the Ethics Boards of the University of Alberta (Pro00015728) and Simon Fraser University (2010s0554), and participants provided prior written informed consent.

Questionnaire data and saliva samples were collected from 667 undergraduate students (396 females and 271 males). The questionnaire was comprised of 32 questions from the SPQ and 50 questions from the AQ. Each question used a 5-point Likert scale, with response choices ranging from “strongly disagree” to “strongly agree.” The SPQ included seven subscales: (1) Constricted Affect, (2) Social Anxiety, (3) Magical Thinking, (4) Unusual Perceptions, (5) Ideas of Reference, (6) Eccentric Behavior, and (7) Odd Speech, which sum to a total schizotypy score. The AQ included five domains: (1) Social skills, (2) Communication, (3) Attention to Detail, (4) Attention Switching, and (5) Imagination, which sum to a total autism spectrum score.

DNA was extracted from saliva samples through standard phenol-chloroform methods. The SNPs of rs9616915 were tagged with fluorophore-labelled primers and used in TaqMan genotyping with a Roche LightCycler 96 Real-Time PCR machine. Fluorescence data were analyzed under Endpoint Genotyping using LightCycler 96 software, v. 1.1.0.1320.

Genotypes were in Hardy–Weinberg equilibrium (*χ*^2^ = 0.11, *p* > 0.9). Two-way ANOVAs were conducted to simultaneously analyze sex differences and scores on the AQ and SPQ subscales using type II sums of squares. These tests were conducted under three genetic models: CC versus CT versus TT, CC versus CT + TT, and CC + CT versus TT. The results of the omnibus tests were subjected to a 12-fold Bonferroni adjustment (0.5/12 = 0.0042) to account for multiple testing of the 12 AQ and SPQ subscales. Note that the intercorrelation between these subscales means that Bonferroni correction for independent tests is undoubtedly excessively conservative. Tests for which the omnibus tests remained significant after this adjustment were examined post hoc to test for significant effects of sex, genotype, and their interaction. Since these tests were conducted post hoc, after correction of the omnibus effects, they were not subjected to further *p* value adjustment. Statistics were conducted in R version 4.0.4, using the Anova() function from the Companion to Applied Regression library, v3.0.10.

## 3. Results

ANOVAs of the effects of sex and genotype under the three different models show two SPQ subscales for which significant amounts of variation are explained after adjustment for multiple testing (Tables 1 and 2): Magical Thinking under all three of the genetic models and Odd Speech in the case of the (CC + CT) versus TT genotype. Post hoc inspection of the sources of variance shows that sex differences are responsible for the effects on Magical Thinking (Table 3), while both sex and the interaction of sex and genotype contribute significantly to the variance in Odd Speech (Table 3). Females score higher than males on both of these SPQ subscales, and males with the TT genotype averaged higher Odd Speech scores than males with the CC or CT genotypes (Tables 1 and 3). The sex differences found in the Magical Thinking subscales are consistent with findings from previous research [40].

## 4. Discussion

The main result of this study is a significant association between genotypes of the SHANK3 SNP rs9616915 and scores on the SPQ Odd Speech subscale, among males. The combined gene and sex effects were significant for this subscale and for sex differences in the SPQ Magical Thinking subscale.

In previous work, the rs9616915TT genotype has been associated with higher autism risk [21] and reduced expression of SHANK3 mRNA in the human hippocampus [26]. Expression levels of SHANK3 are also affected by prenatal dihydrotestosterone levels in mice [41]. These results are consistent with the sex difference in the effects of SHANK3 genotype on scores for Odd Speech and the strong male bias in autism itself.

The SPQ Odd Speech subscale measures tendencies to ramble, digress, jump illogically between subjects, and forget what one is trying to say [42]. The association of rs9616915 genotype with Odd Speech thus suggests that this SNP affects aspects of speech and language in healthy human populations. This finding is concordant with three lines of additional evidence: (1) PMS considered as primarily a speech and language disorder [34] with core phenotypes mediated by disruption to white matter tracts underlying language [43]; (2) the presence of prominent speech alterations in the three psychiatric disorders linked with SHANK3, autism, schizophrenia, and bipolar disorder [33, 44, 45]; and (3) vocalization alterations found in SHANK3-deficient animal models [30, 36, 37].

Haploinsufficiency of SHANK3 is the main genetic cause of PMS [46]. PMS is characterized by autism, language impairments, and intellectual disability [10]. However, autism in PMS is sometimes atypical because many subjects show relatively low levels of the repetitive behaviors and intolerance to change that are characteristic of ASD [8, 34]; for example, individuals with PMS who do not meet an autism diagnosis by the ADI-R usually show subthreshold levels for restricted interests and repetitive behavior [8]. Ponson et al. [34] also described evidence that autistic behaviors in PMS are caused by somewhat different neurological networks than conventional ASD and, therefore, that PMS may be especially a disorder of language.

Neurological phenotypes of PMS are characterized by disruption to long white matter tracts in the brain that underlie core features of speech [43]. SHANK3-deficient PMS patients have been found to have alterations in the inferior frontooccipital fasciculus (IFOF) and the uncinate fasciculus (UF) [43]. The IFOF is associated with semantic processing, reading, and basic naming [47], while the UF influences language comprehension, long-term memory, and general speech performance [48]. Identification of disruption to specific speech-associated long white matter tracts in PMS provides a possible neurological basis for the observed language phenotypes and supplies a point of comparison between PMS and other psychiatric disorders with speech dysfunction.

In addition to PMS, structural alterations to the IFOF and UF are also found in bipolar disorder [49, 50], schizophrenia [51, 52], and ASD [53, 54]. These disorders each have altered language phenotypes: mania in bipolar disorder involves speech that is “pressured” and difficult to understand [45]; schizophrenia is characterized by communication with loose associations, lack of logic, and frequently shifting topics [44, 55]; and autism typically involves delayed and altered development of speech and language [33].

Animal models provide further insight into the relationship of SHANK3 with speech and language. Thus, SHANK3-deficient mutant macaques, rats, and mice exhibited reduced vocalization and altered auditory phenotypes [36, 37], and mice additionally showed increased pitch discrimination [30]. Delayed or absent language and absolute pitch are also commonly found in humans with autism [31, 56]. The related vocalization and auditory-related phenotypes of SHANK3-deficient animal models and humans with ASD provide intriguing indications that SHANK3 is linked to communication in animals and humans.

This study has several limitations. First, only one SNP was analyzed. The rs9616915 SNP was selected because it has been linked to autism [21] and to SHANK3 expression in the hippocampus [26]. Additional SNPs for use in future work could be chosen across the gene. Second, the study population included only Caucasian undergraduate students. Inclusion of other populations would be useful for comparison and to be more representative of the general population. Third, we used the AQ and SPQ questionnaires that quantify only a few speech- and language-related phenotypes. Future studies of SHANK3 SNPs should use questionnaires specific to speech and language phenotypes to focus more closely and specifically on how these traits vary among typical individuals with different SHANK3 genotypes. Based on our results, imaging genetic and speech-language studies of typical males carrying different genotypes of rs9616915 in particular should provide useful insights into the neurological and psychological bases of speech and language alterations and autism among individuals with SHANK3 mutations and Phelan–McDermid syndrome.

## Figures and Tables

**Table 1:** Scales and subscales by sex and genotype.

Scale	Males			Females		
	CC (*N* = 68)	CT (*N* = 144)	TT (*N* = 59)	CC (*N* = 97)	CT (*N* = 185)	TT (*N* = 114)
	Mean ± SD	Mean ± SD	Mean ± SD	Mean ± SD	Mean ± SD	Mean ± SD
AQ Social Skills	1.81 ± 1.81	2.28 ± 2.05	2.29 ± 2.24	2.45 ± 2.31	2.51 ± 2.19	2.51 ± 2.45
AQ Attention Switching	4.59 ± 2.00	4.94 ± 2.05	5.00 ± 1.70	4.98 ± 2.10	5.04 ± 2.02	4.99 ± 2.11
AQ Attention to Detail	5.53 ± 2.30	5.54 ± 2.12	5.25 ± 1.83	5.44 ± 2.02	5.62 ± 2.23	5.32 ± 2.04
AQ Communication	1.87 ± 1.43	2.41 ± 1.83	2.19 ± 1.78	2.57 ± 1.77	2.45 ± 1.98	2.44 ± 1.78
AQ Imagination	2.57 ± 1.61	2.56 ± 1.62	2.36 ± 1.41	2.16 ± 1.50	2.07 ± 1.49	2.09 ± 1.61
AQ total	16.37 ± 5.01	17.74 ± 5.50	17.08 ± 5.34	17.61 ± 5.36	17.68 ± 5.88	17.35 ± 6.08
SPQ Ideas of Reference	15.99 ± 3.73	16.60 ± 4.48	16.90 ± 3.90	17.41 ± 4.30	17.34 ± 4.18	17.26 ± 4.60
SPQ Constricted Affect	15.63 ± 4.75	15.82 ± 4.92	15.54 ± 4.26	14.68 ± 4.99	14.68 ± 5.01	15.18 ± 5.07
SPQ Eccentric Behavior	12.07 ± 3.93	12.10 ± 3.60	12.07 ± 4.01	12.03 ± 3.81	11.64 ± 3.74	11.75 ± 4.09
SPQ Social Anxiety	11.32 ± 3.93	11.55 ± 3.58	11.47 ± 3.71	11.94 ± 3.99	12.04 ± 4.15	12 ± 4.49
SPQ Magical Thinking	7.50 ± 3.88	7.264 ± 3.44	6.69 ± 2.53	8.36 ± 3.43	8.78 ± 3.72	8.25 ± 3.64
SPQ Odd Speech	12.57 ± 3.08	12.82 ± 2.76	13.76 ± 2.81	13.90 ± 2.93	13.71 ± 2.98	13.48 ± 3.26
SPQ Unusual Perception	10.54 ± 2.74	10.75 ± 2.69	10.54 ± 2.69	10.45 ± 3.00	10.21 ± 2.78	10.14 ± 2.89
SPQ total	85.63 ± 16.14	86.90 ± 15.00	86.98 ± 14.01	88.77 ± 15.43	88.4 ± 16.12	88.06 ± 17.93

**Table 2:** Results from 2-way ANOVA on sex and genotype, for the three genotypic models.

Scale	CC versus CT versus TT	(CC + CT) versus TT	(CT + TT) versus CC
	*F*_(5,661)_, *p*	*F*_(3,663)_, *p*	*F*_(3,663)_, *p*
AQ Social Skills	1.21, 0.30	1.28, 0.28	2.02, 0.11
AQ Attention Switching	0.52, 0.76	0.38, 0.77	0.85, 0.46
AQ Attention to Detail	0.45, 0.82	0.60, 0.61	0.04, 0.99
AQ Communication	1.51, 0.19	1.04, 0.37	2.3, 0.076
AQ Imagination	2.57, 0.026	4.21, 0.005821	4.05, 0.0072
AQ total	0.71, 0.62	0.27, 0.85	0.92, 0.43
SPQ Ideas of Reference	1.53, 0.18	2.24, 0.08	2.49, 0.060
SPQ Constricted Affect	1.25, 0.28	2.07, 0.10	1.81, 0.14
SPQ Eccentric Behavior	0.36, 0.88	0.38, 0.77	0.58, 0.63
SPQ Social Anxiety	0.59, 0.71	0.93, 0.43	0.98, 0.40
SPQ Magical Thinking	**5.25, 0.000098**	**8.40, 0.000018**	**7.86, 0.000037**
SPQ Odd Speech	3.27, 0.0063	**5.28, 0.0013**	3.90, 0.0088
SPQ Unusual Perception	0.89, 0.49	1.24, 0.30	1.39, 0.25
SPQ total	0.50, 0.78	0.72, 0.54	0.82, 0.48

Bonferroni significant results are shown in boldface.

**Table 3:** Sources of variation for the Bonferroni-adjusted significant results in Table 2.

Source	SS	Df	*F*	*p*
*(a) Magical Thinking*				
*Sex by (CC versus CT versus TT) genotype*				
Sex	296.5	1	23.76	0.0000014
Genotype	31.2	2	1.24	0.29
Genotype *x* sex	13.4	2	0.54	0.58
Residuals	8247.4	661		

*(b) Magical Thinking*				
*Sex by (CC* *+* *CT) versus TT genotype*				
Sex	295.5	1	23.71	0.0000014
Genotype	28.9	1	2.32	0.13
Genotype *x* sex	2.1	1	0.17	0.68
Residuals	8261.1	663		

*(c) Magical Thinking*				
*Sex by (CT* *+* *TT) versus CC genotype*				
Sex	283.0	1	22.66	0.0000024
Genotype (CT + TT) versus CC	0.2	1	0.01	0.91
Genotype *x* sex	11.5	1	0.92	0.34
Residuals	8280.3	663		

*(d) Odd Speech*				
*Sex by (CC* *+* *CT) versus TT genotype*				
Sex	81.4	1	9.20	0.0025
Genotype	4.4	1	0.49	0.48
Genotype *x* sex	50.7	1	5.74	0.0168
Residuals	5861.3	663		

## Data Availability

The data used to generate the findings of this study are available from the corresponding author upon request.
